# Identification of a newly isolated lytic bacteriophage against K24 capsular type, carbapenem resistant *Klebsiella pneumoniae* isolates

**DOI:** 10.1038/s41598-020-62691-8

**Published:** 2020-04-03

**Authors:** Marianna Horváth, Tamás Kovács, Sarshad Koderivalappil, Hajnalka Ábrahám, Gábor Rákhely, György Schneider

**Affiliations:** 1 Department of Medical Biology and Central Electron Microscope Laboratory, University of Pécs, Medical School, Pécs, Hungary; 2 Department of Biotechnology, Nanophagetherapy Center, Enviroinvest Corporation, Pécs, Hungary; 3 Department of Biotechnology, University of Szeged, Szeged, Hungary; 4 Institute of Biophysics, Biological Research Center, Szeged, Hungary; 5 Department of Medical Microbiology and Immunology, University of Pécs, Medical School, Pécs, Hungary

**Keywords:** Bacteriophages, Bacterial infection

## Abstract

The increasing incidence of carbapenemase-producing *K. pneumoniae* strains (CP-Kps) in the last decade has become a serious global healthcare problem. Therapeutic options for the treatment of emerging hospital clones have drastically narrowed and therefore novel approaches must be considered. Here we have isolated and characterized a lytic bacteriophage, named vB_KpnS_Kp13, that was effective against all Verona integron-encoded metallo-β-lactamase (VIM) producing *K. pneumoniae* isolates originating from hospital samples (urine, blood, sputum and faeces), belonging to the ST15 clonal lineage and expressing the K24 capsule. Morphological characterization of vB_KpnS_Kp13 showed that the newly identified phage belonged to the *Siphoviridae* family, and phylogenetic analysis showed that it is part of a distinct clade of the *Tunavirinae* subfamily. Functional analysis revealed that vB_KpnS_Kp13 had relatively short latent period times (18 minutes) compared to other *K. pneumoniae* bacteriophages and could degrade biofilm by more than 50% and 70% in 24 and 48 hours respectively. Complete *in vivo* rescue potential of the new phage was revealed in an intraperitoneal mouse model where phages were administered intraperitoneally 10 minutes after bacterial challenge. Our findings could potentially be used to develop specific anti-CP-Kps bacteriophage-based therapeutic strategies against major clonal lineages and serotypes.

## Introduction

*Klebsiella pneumoniae* is one of the most important opportunistic nosocomial pathogens, able to colonize the skin and mucosae of humans and cause urinary tract infections, septicaemia and pneumonia, predominantly in immunocompromised individuals^1,2^. In hospitals, colonization leads to patients becoming reservoirs and a source of cross infection for other patients^3^.

Dissemination of antibiotic resistance, particularly to carbapenem, is associated with highly diverse isolates of *K. pneumoniae*. In this process the importance of certain clonal groups (CGs) such as CG258 and CG15 has been demonstrated. These CGs contain several high-risk international clones including ST11, ST258 and ST15^4,5^. Although recent studies have not described ST15 as a widespread clonal lineage^6^, other data indicates that ST15 is a pan-drug resistant widespread clone, frequently associated with carbapenemase production^7^ and nosocomial infections. ST15 was recently identified in the USA^8^, Brazil^9^, China^10,11^, Taiwan^12^, and several European countries^13^ including Portugal^14^, Belgium^15^, Bulgaria^16^ and Hungary^4^. Some epidemiological surveys suggested that the K24 is a frequent capsular type linked to ST15^9,17,18^.

One potential option to combat these pathogens is to use bacteriophages as they have well defined target spectrums, characterized by host range specificity^19^. This feature makes them suitable therapeutic agents without influencing the normal microbiota of the patient^20^. Certain bacteriophages also have the capacity to target and destroy defined capsular types and weaken biofilm typically produced by *Klebsiella pneumoniae*^21^.

The most important virulence factors of *K. pneumoniae* are the capsular polysaccharides (CPS) and lipopolysaccharides (LPS) typically used for K-, and O-serotyping respectively. Over 80 capsular types have been defined, including 77 types from reference strains identified between 1926 and 1977 using serological tests, and 5 new types (KN1 to KN5) characterized by molecular genotyping and structural analyses in recent years^22–24^.

Here we isolated and characterized a lytic bacteriophage (vB_KpnS_Kp13), which was effective against all the carbapenemase-producing *Klebsiella pneumoniae* strains (CP-Kps) originating from the same teaching hospital and belonging to ST15. Phage vB_KpnS_Kp13 was specific for K24 capsular type. The K24 serotype was recently reported to be frequently associated with ST15^9,17,18^ and frequently reported from other CP-Kps^25^. Our results may aid the development of anti CP-Kps bacteriophage-based therapeutic strategies targeting major clonal lineages.

## Results

### Morphology, host range and efficiency of plating (EOP)

Altogether 13 bacteriophages were isolated from wastewater against *K. pneumoniae* hospital isolates. Four of them were effective against all *Klebsiella pneumoniae* isolates with the K24 capsule. Based on their restriction profiles (*Eco*RI and *Hind*III; data not shown) one proved to be distinct from the other three and this single one always showed a clear lytic zone (around 10 mm diameter) without the emergence of phage-resistant colonies and showed a characteristic wide halo (~ 4 mm) (Supplementary Fig. 1). This phage was vB_KpnS_Kp13 and characterized in this study.

**Figure 1:**
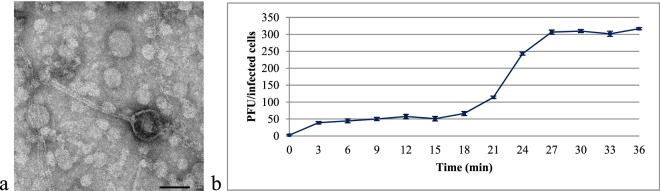
Characteristics of vB_KpnS_Kp13. (**a)** Electron micrograph of phage vB_KpnS_Kp13 shows the typical features of the *Siphoviridae* family. Phage was stained with 1.5% w/v phospho-tungstic acid. Scale bar represents 50 nm. (**b)** One-step growth curve of phage vB_KpnS_Kp13. The plaque forming unit (PFU) per infected cell at different times are shown. Data are the mean of 3 independent experiments. Error bars represent standard deviation.

Transmission electron microscopy (TEM) analysis indicated that phage vB_KpnS_Kp13 comprised a ~60 nm diameter head and a flexible, non-contractile, ~200 nm tail (Fig. 1a). Based on these traits, phage vB_KpnS_Kp13 was classified as *Caudovirales* in the *Siphoviridae* family^26^.

Host range tests with 89 *Klebsiella spp*. isolates showed that phage vB_KpnS_Kp13 was only effective on *K. pneumoniae* isolates with the K24 capsule, causing lysis in 100% (40/40) of these strains (Table 1). Other *Klebsiella spp*. isolates with either known (K1, K2, K3, K6, K9, K11, K12, K13, K17, K19, K20, K21, K24, K27, K30, K33, K47, K52 and K64) or unknown, but non-K24 capsular types were not sensitive to vB_KpnS_Kp13.

**Table 1:** Host range of vB_KpnS_Kp13 tested on *Klebsiella spp*. isolates.

Bacterial strain	Number of isolates	Sequence type	K-serotype	Lysis	Reference/Source
*Klebsiella pneumoniae* subsp. *pneumoniae*	1	ST23	K1	−	NTUH-K2044
*Klebsiella pneumoniae* subsp. *pneumoniae*	1	ST66	K2	−	CIP 52.145*
*Klebsiella pneumoniae* subsp. *rhinoscleromatis*	1	ND	K3	−	CIP 80.51*
*Klebsiella quasipneumoniae*	1	ST489	K6	−	ATCC 700603
*Klebsiella pneumoniae* subsp. *pneumoniae*	1	ND	K9	−	CIP 52.207*
*Klebsiella pneumoniae* subsp. *pneumoniae*	1	ND	K11	−	CIP 52.215*
*Klebsiella pneumoniae* subsp. *pneumoniae*	1	ND	K12	−	CIP 52.216*
*Klebsiella pneumoniae* subsp. *pneumoniae*	1	ND	K13	−	CIP 52.217*
*Klebsiella pneumoniae* subsp. *pneumoniae*	1	ND	K17	−	CIP 52.221*
*Klebsiella pneumoniae* subsp. *pneumoniae*	1	ND	K19	−	CIP 52.223*
*Klebsiella pneumoniae* subsp. *pneumoniae*	1	ND	K20	−	CIP 52.224*
*Klebsiella pneumoniae* subsp. *pneumoniae*	1	ND	K21	−	CIP 52.225*
*Klebsiella pneumoniae* subsp. *pneumoniae*	1	ST59	K24	+	CIP 52.229*
*Klebsiella pneumoniae* subsp. *pneumoniae*	39	ST15	K24	+	Clinical isolates, CCH**
*Klebsiella pneumoniae*	30	ND	not K24	−	Clinical isolates, own collection
*Klebsiella pneumoniae* subsp. *pneumoniae*	1	ND	K27	−	CIP 52.232*
*Klebsiella pneumoniae* subsp. *pneumoniae*	1	ND	K30	−	CIP 52.235*
*Klebsiella pneumoniae* subsp. *pneumoniae*	1	ND	K33	−	CIP 53.8*
*Klebsiella pneumoniae* subsp. *pneumoniae*	1	ND	K47	−	CIP 53.23*
*Klebsiella pneumoniae* subsp. *pneumoniae*	1	ST38	K52	−	MGH 78578
*Klebsiella pneumoniae* subsp. *pneumoniae*	1	ND	K64	−	CIP 80.47*

Details of each bacterial strains are reported (+: clear plaque formation; −: no plaque formation; ND: not defined).

*Strains with CIP prefixes were purchased from the Institut Pasteur (France).

**CCH: Countess of Chester Hospital, Department of Microbiology, Chester, Cheshire, UK.

The EOP analysis revealed a high productive (EOP ≥ 0.5) infection of phage vB_KpnS_Kp13 for 37 of the 40 (92.5%) sensitive strains. Medium production occurred in 3 of the 40 (7.5%) strains sensitive to vB_KpnS_Kp13 in the double-layer agar (DLA) assay (Supplementary Table 1).

### One-step phage growth experiments – burst-size determination

The one-step growth experiment was performed to determine the latent time period and burst size of the phage. A triphasic curve was obtained showing the latent period, log or rise period, and plateau period. The phage vB_KpnS_Kp13 showed relatively short latency period (18 min) followed by a rise period of 10 min and a growth plateau starting at 27 min (Fig. 1b). The burst size of vB_KpnS_Kp13 was ~220 phage particles per infected bacteria.

### Killing dynamic of vB_KpnS_Kp13 against *K. pneumoniae* 533 strain and phage-resistant clone screening

**Figure 2:**
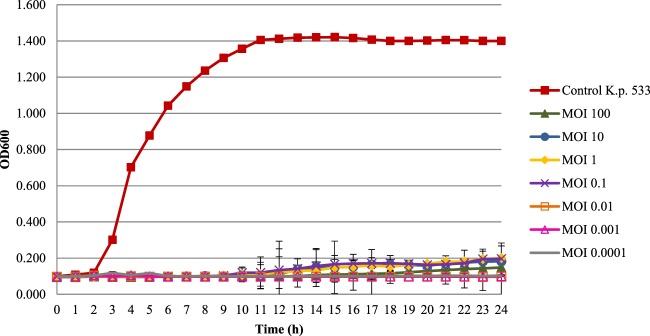
Time-kill assay of phage vB_KpnS_Kp13 against *K. pneumoniae* 533. Overnight bacterial culture was diluted (10^5^ CFU/ml) and infected with phage vB_KpnS_Kp13 at MOI of 100 (green, ▲), at MOI of 10 (blue, ●), at MOI of 1 (yellow, ◆), at MOI of 0.1 (purple, ×), at MOI of 0.01 (orange, □), at MOI of 0.001 (pink, Δ) and at MOI of 0.0001 (grey). Non-phage treated bacterial cultures (control *K.p*. 533, red, ■) were used as a positive control. Optical density at 600 nm was measured every hour up to 24 h. Each data point is the mean from 3 experiments. Standard deviations (±SD) are shown as vertical lines.

Phage vB_KpnS_Kp13 inhibited the proliferation of *K. pneumoniae* 533 in a concentration dependent manner. At multiplicity of infection (MOI) between 0.0001 and 100, phage vB_KpnS_Kp13 effectively inhibited the growth of *K. pneumoniae* 533 in LB broth for 24 h (Fig. 2).

Several spot testing of the phage vB_KpnS_Kp13 on the lawn of different *Klebsiella pneumoniae* isolates, indicated that no resistant bacterium clones emerged against the newly isolated phage. In the liquid culture screening at different MOIs, we could not isolate phage-resistant clones.

### Biofilm degradation assay and Confocal Laser Scanning Microscopy (CLSM) analysis

**Figure 3:**
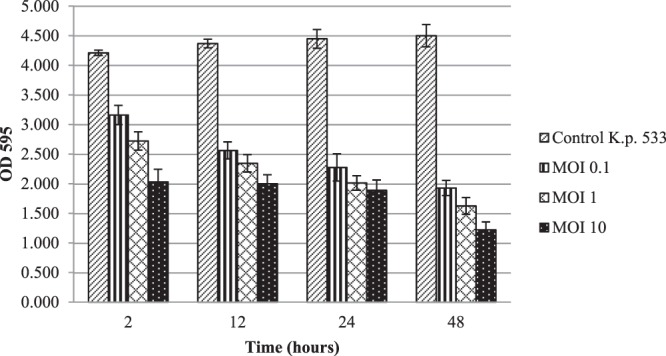
Effect of vB_KpnS_Kp13 on *K. pneumoniae* 533-produced biofilm degradation. The established biofilm was infected with vB_KpnS_Kp13 at MOI of 0.1 (striped), at MOI of 1 (gridded) and MOI of 10 (spotted). Non-phage treated biofilm (side striped) was used as a positive control. OD at 595 nm was measured at 2, 12, 24 and 48 h. Each data point is the mean from 3 experiments. Error bars represent standard deviation.

The *K. pneumoniae* 533 isolate is a strong biofilm producer (mean OD = 4.4 ± 0.1) based on the formerly established OD cut-off (ODc) values^27^. Here, pre-established biofilms were degraded by phage vB_KpnS_Kp13 in a MOI and time dependent manner. A 51.8% loss in biomass was detected after 2 h incubation at MOI 10. Biomass reduction was further increased to 54.2%, 57.5%, and 72.9% after 12, 24 and 48 h respectively if compared to the OD values of the untreated biofilm control. These values were also reduced if the applied phage:bacterium ratios were 1:1 (MOI 1) and 1:10 (MOI 0.1), but still led to marked biofilm degradation (Fig. 3).

**Figure 4:**
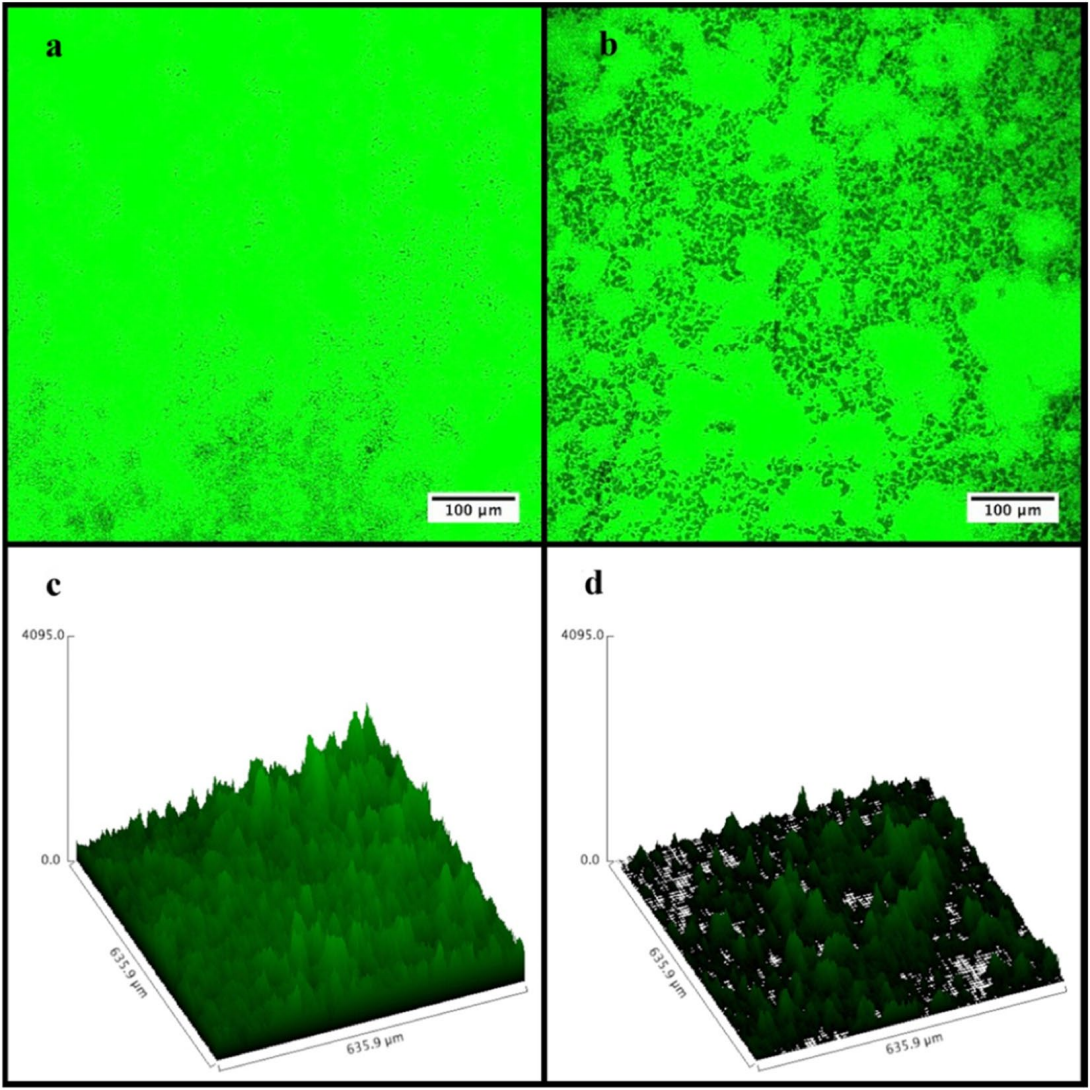
Visualization of *Klebsiella pneumoniae* 533 biofilm by CLSM. (**a**) Control (only bacterium, no phage). (**b**) Treated with phage vB_KpnS_Kp13. Scale bar represents 100 µm. Surface plot was generated using CLSM microscopy software (Olympus FV1000). (**c**) Control *K. pneumoniae* 533 biofilm surface plot. (**d**) Phage vB_KpnS_Kp13-treated 533 biofilm surface plot.

Biofilm degradation efficacy of phage vB_KpnS_Kp13 was also analysed with Confocal Laser Scanning Microscopy (CLSM). CLSM images showed that phage vB_KpnS_Kp13 firmly reduced the biomass and thickness of the biofilm architecture (Fig. 4a,b). Confocal micrographs show the non-infected control (only bacterium, no phage) displayed a highly structured matrix formation (Fig. 4c). The phage vB_KpnS_Kp13-treated biofilm showed disintegrated clumps with scattered microflora that resulted in the collapse of the pre-formed biofilm (Fig. 4d). The structural difference in the control biofilm and phage-treated biofilm, showed the efficacy of phage vB_KpnS_Kp13 on biofilm degradation. The biomass of the control *K. pneumoniae* 533 biofilm was 13.12 μm^3^/μm^2^, in contrast to the vB_KpnS_Kp13 treated biofilm where this value was 4.76 μm^3^/μm^2^ (Supplementary Table 2).

### Genome characteristics

**Figure 5:**
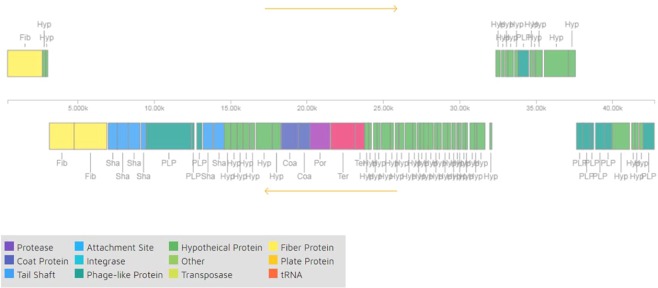
Linear genomic map of vB_KpnS_Kp13 obtained with PHASTER. Different functional groups of phage vB_KpnS_Kp13 are denoted by different colors, according to their function.

The phage vB_KpnS_Kp13 genome measured 43,094 bp (accession number MK170446) with a 50.6% G + C content. It contains 75 predicted open reading frames (ORFs), while no tRNA gene was predicted by GeneMark program. The orientation of genome annotation showed that 61 genes are on the minus strand and 14 are on the plus strand. A linear genome map of phage vB_KpnS_Kp13 was obtained from a PHASTER analysis (Fig. 5).

The ORF analysis (GeneMark) of the complete vB_KpnS_Kp13 genome revealed 5 major functional clusters^21^, hypothetically responsible for coding determinants of (i) DNA replication/modification/transcriptional regulations: DNA N-6-adenine methyltransferase (ORF72), ssDNA-binding protein (ORF1), DNA terminase (large subunit = ORF24 and small subunit = ORF 25), (ii) structure and packaging: portal protein (ORF23), major capsid protein (ORF21), membrane protein (ORF32 and ORF73) and head morphogenesis protein (ORF22), (iii) host lysis: holin (ORF65), endolysin (ORF64), (iv) tail structure: tail fiber proteins (ORF4, ORF5 and ORF6), tail assembly (ORF10) and tail length tap measure proteins (ORF9) and (v) hypothetical or unknown functions.

The hypothetical capsule depolymerase gene, responsible for the degradation of the K24 capsule, was encoded by ORF2 (2289 bp), that shared different rates of amino-acid sequence similarities to other known phage depolymerases: *Klebsiella* phage KOX1 (77.67%), *Klebsiella* phage JY917 (75.23%), *Klebsiella* phage KP36 (68.08%), *Klebsiella* phage GH-K3 (66.54%), *Klebsiella* phage KLPN1 (58.13%), *Klebsiella* phage Sushi (46.32%) and *Klebsiella* phage K5-2 (31.82%).

### Phylogenetic relations

**Figure 6:**
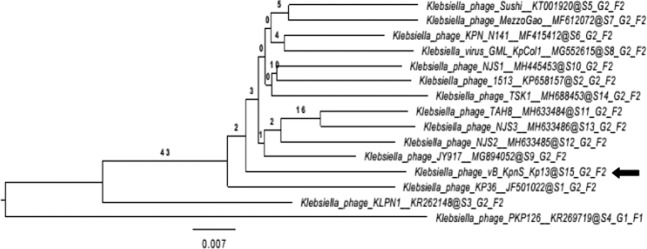
Whole genome based phylogenetic relations of phage vB_KpnS_Kp13 generated with VICTOR. The bacteriophages and their accession numbers are: MezzoGao (MF612072.1), KpCol1 (MG552615.1), N141 (MF415412.1), Sushi (KT001920.1), TAH8 (MH633484.1), NJS3 (MH633486.1), NJS2 (MH633485.1), NJS1 (MH445453.1), 1513 (KP658157.1), KP36 (JF501022.1), KPK126 (KR269719.1), KLPN1 (KR262148.1), JY917 (MG894052.1), TSK1 (MH688453) and vB_KpnS_Kp13 (MK170446).

A phylogenetic analysis of the complete vB_KpnS_Kp13 genome was performed using VICTOR web service. It was found that the *Klebsiella* JY917 phage is the closest relative of vB_KpnS_Kp13 with 90% similarity (Fig. 6). The Genome BLAST Distance Phylogeny (GBDP) tree inferred using the formula D4 and yielding an average support of 3%. This data is consistent with the obtained and current International Committee on Taxonomy of Viruses (ICTV) taxonomy, confirming that vB_KpnS_Kp13 forms a distinct clade of the *Tunavirinae* subfamily and *Kp36virus* genus.

### Mouse intraperitoneal (IP) phage rescue model

The therapeutic potential of vB_KpnS_Kp13 was revealed in an intraperitoneal (IP) mouse model where the effect of vB_KpnS_Kp13 against *K. pneumoniae* 533 was strongly dependent on the time that passed between the bacterial infection and phage administration.

**Figure 7:**
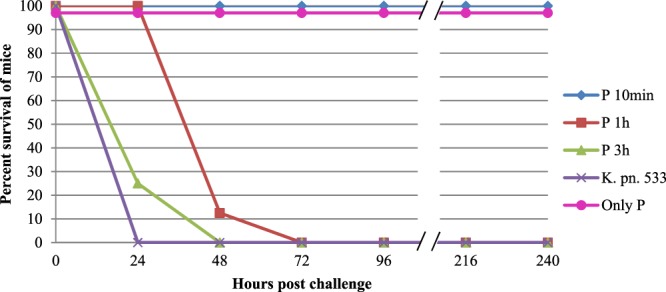
Therapeutic effect of vB_KpnS_Kp13 in an intraperitoneal (IP) mouse infection model. Phage administration occurred 10 min (P 10 min, blue, ♦), 1 hour (P 1 h, red, ■) and 3 hours (P 3 h, green, ▲) post-infection, while the *K. pneumoniae* 533 (*K. pn*. 533, purple, ×) and the phage vB_KpnS_Kp13 (only P, pink, ●) suspensions were used as positive and negative controls, respectively. The graphs show percent survival of mice from the start of IP infection over 240 h. Each data point is the mean from two experiments (altogether 8 mice in one group). Error bars represent standard deviation. For better visibility we artificially lowered the values of the phage control (only P, pink, ●) from 100% to 97%.

In the positive control group, 8 mice were challenged with 2 × 10^8^ CFU of *K. pneumoniae* 533 in suspension per mouse, and all of them died within 24 h (LD_50_ = 12 h). No effect was observed when the phage suspension (1.75 × 10^8^ PFU/mice) was administered to uninfected mice (negative control group, n = 8). Phage administration within 10 min of bacterial challenge prevented death in all mice (n = 8), and by this assuring 100% survival without showing any signs of infection. This therapeutic effect was not observed if the phage suspension was administered 1 h after bacterial challenge. In this case, the survival rate was still 100% at 24 h, but the general condition of the mice drastically deteriorated, and their survival decreased to 12.5% (1/8 mice) 48 h after infection. No rescuing effect of vB_KpnS_Kp13 was detected if it was administered 3 h after bacterial challenge (Fig. 7). Ten days after bacterial challenge and subsequent phage administrations, surviving mice were sacrificed and active phage particles were isolated from the lungs, spleen and blood. No active phage particles were detected.

## Discussion

The appearance and dissemination of carbapenem-resistant *Klebsiella pneumoniae* isolates in hospital environments is one of the biggest challenges of today’s healthcare system. Therapeutic options are limited, with the aim being to treat colonized patients and remove endemic strains from the hospital environment. Due to their high specificity and antibacterial features, bacteriophages are promising candidates for these tasks^25^.

The antibacterial potential of the newly isolated phage vB_KpnS_Kp13 against the clonally disseminated, VIM carbapenemase-producing *Klebsiella pneumoniae* strains (CP-Kps), possessing the K24 capsule in an English Tertiary Hospital demonstrated the viability of phage vB_KpnS_Kp13 as a possible alternative method to manage this local problem.

Phage vB_KpnS_Kp13 was characterized by several favourable and unique features. Notably, no phage-resistant bacterium clones emerged in this study, a problem that can lead to therapeutic failure during phage therapy^28^. DNA N-6-adenine methyl transferase, encoded by ORF72 (729 bp), might be a useful feature of phage vB_KpnS_Kp13 since it could protect the injected phage DNA against bacterial nucleases^29^. It is unknown if this gene is functional in vB_KpnS_Kp13, thereby supporting phage infection. Similar methyltransferases have been described from the Sushi (98%), Kp1513 (98%) and JY917 (97%) *K. pneumoniae* phages, but no data were available concerning the resistance ratios against these phages. The other crucial aspect of the phage action is the receptor itself. Although we currently have no data about the receptor of vB_KpnS_Kp13, lack of resistance against this bacteriophage strongly suggests that target of this phage contributes to survival of strain 533. Due to its seemingly specific action on K24 capsular type *K. pneumoniae* isolates, it was reasonable to question if K24 was the only receptor of vB_KpnS_Kp13 or if another surface associated structure was also necessary. Recent studies showed that capsule itself could be the receptor itself^30^. In contrast spontaneous capsule mutants of *K. pneumoniae* 533 remained sensitive to vB_KpnS_Kp13. Receptor structure of vB_KpnS_Kp13 is still under investigation, but similarly to other previously reported *K. pneumoniae* bacteriophages KP34^31^ and KP 1513^32^, our phage also showed a narrow host range. Therefore, it could be applied in phage therapy in combination with other bacteriophages or to treat local spread of K24 *K. pneumoniae*.

Inability of vB_KpnS_Kp13 to lyse and/or weaken the lawn of other non-K24 serotype *K. pneumoniae* strains (Table 1) referred to the specific feature of the capsule depolymerase encoded by ORF2 of vB_KpnS_Kp13. Only one study focuses on the functional analysis of a K24 specific phage^33^. Beyond that another bacteriophage (K5-2) was recently reported as lytic against a K5 serotype *K. pneumoniae* strain by forming a characteristic halo and showing different rates of lytic activity against 7 other serotypes including K24 and K30^34^. Lack of marked sequence homology on both genomic and protein level and – in contrast to K5-2 – inability of vB_KpnS_Kp13 to weaken K30 (Table 1) suggest the different origins of the two capsule depolymerases of K5-2 and vB_KpnS_Kp13. The K24 depolymerase of vB_KpnS_Kp13 was highly specific with limited homologies to other known capsule depolymerases.

The latent period time (18 min) of phage vB_KpnS_Kp13 was longer that of *Klebsiella* phage φBO1E (*Podoviridae*; 10 min)^35^. The burst size of vB_KpnS_Kp13 (~220 phage particles/infected cell) was not extremely high compared with two phages in the *Siphoviridae*^36^ and *Podoviridae*^35^ families, but they assured an effective bactericidal potential as vB_KpnS_Kp13 could prevent proliferation of its target bacterium from an MOI of 0.01. Compared to phage TSK1^21^, this value was lower by 2 orders of magnitude and therefore was more effective. There is no relevant efficacy studies published *in vivo* or *in vitro* using *K. pneumoniae* phages such as JY917 (accession MG894052.1), MezzoGao (accession MF612072.1), Sushi (accession KT001920.1), NJS1 (accession MH445453.1), 1513 (accession KP658157.1), KP36 (accession JF501022.1), and NJR15 (accession MH633487.1). vB_KpnS_Kp13 has shown its characteristic antibacterial potential without the emergence of resistant mutants during the one-week test periods.

To best of our knowledge this is the first described phage active against K24 producing *K. pneumoniae* strains. A characteristic biofilm degradation potential was also visualized by the classical assay and Confocal Laser Scanning Microscopy (CLSM). Few works detailing biofilm degradation are available, but similarly to phage Z^36^ and TSK1^21^, the phage vB_KpnS_Kp13 showed drastically degraded biofilm, reducing the biomass by ~73% 48 h post-treatment. Analysis of biofilm formation is essential as it is an important survival strategy of bacteria on biotic and abiotic surfaces, potentially inhibiting antibiotic efficacy and protecting bacteria from host responses^21^. The biofilm disruptor capacity of vB_KpnS_Kp13 could solve this problem in the future.

Rescue experiments with vB_KpnS_Kp13 showed the potency of this phage *in vivo*. Survival of 100% of the mice, if treatment occurred 10 min after bacterial challenge, indicated the efficacy of vB_KpnS_Kp13 *in vivo*. Failure of the 1 h treatment suggested that 533 gained advantage and by this vB_KpnS_Kp13 treatment could only delay the death of the experimental animals. These results were comparable with the relevant (MOI 1) results of a recent study^37^ where however a slowly escalating wound infection model was investigated using the K2 serotype *K. pneumoniae* strain B5055 and the lytic phage Kpn5. Effective therapeutic potential was also demonstrated with phage SS^38^ but reduction of colonization of the *K. pneumoniae* strain B5055 in the lungs could only be reached if MOI 100 was applied. From that point the efficacy of vB_KpnS_Kp13 was revealed in such a mouse model where bacterial challenge caused the death of infected mice in 24 hours without phage treatment.

## Conclusion

Phage vB_KpnS_Kp13 was effective against the locally disseminated *Klebsiella pneumoniae* isolates, possessing the K24 capsule type and belonging to the ST15 clonal linage. As this phage targeted a high-risk international clone our results may aid the development of anti-CP-Kps bacteriophage-based therapeutic strategies targeting major clonal lineages.

## Materials and methods

### Bacterial strains and growth conditions

The VIM-producing carbapenem-resistant clinical *Klebsiella pneumoniae* 533 isolate belonging to the ST15 clonal lineage and expressing the K24 capsule was used for bacteriophage isolation and for phage propagation. All ST15 K24 strains were isolated from the Microbiology Laboratories of the University Hospital in Chester, England. Samples from urine, blood, sputum and faeces were collected from different wards (Supplementary Table 3).

For phage host range testing an additional 88 *K. pneumoniae* and 1* K. quasipneumoniae* isolates were used (Supplementary Table 3). All bacterial strains were grown under aerobic conditions in Luria-Bertani (LB) broth or agar (1.5%) at 37 °C.

### Phage isolation and purification

*Klebsiella pneumoniae*-specific bacteriophages were isolated from sewage samples from the local wastewater treatment station (Pellérd, Hungary). Briefly, 10 ml sewage was added to 200 ml log phase *K. pneumoniae* 533 culture and incubated at 37 °C for 24 h shaking at 120 RPM. Residual bacterial cells were removed with centrifugation (5000 RPM, 8 min) and supernatant was sterile filtered (0.2 µm pore size filter; Sarstedt, Germany). The phage suspensions were serially diluted in phosphate-buffered saline (PBS) and purified in two consecutive steps by using the double-layer agar (DLA) technique^39^ with 0.4% agar. The resulting high titre (10^9^ PFU/ml) phage stocks were stored at +4 °C and used for further characterization.

### Transmission electron microscopy (TEM)

One drop from the purified high titre (10^9^ PFU/ml) phage stock was deposited onto formvar-coated copper grids (Pelco Grids, Canada) and negatively stained with 1.5% w/v phospho-tungstic acid (Merck, Germany). Phages were examined using a JEM-1400 Flash TEM (JEOL USA Inc.) transmission electron microscope operated at 120 kV acceleration voltage. Based on their morphology, phages were classified according to the guidelines of the International Committee on Taxonomy of Viruses (ICTV)^40^.

### Phage host range testing and K24 specific PCR

Spot testing^41^ of the purified phage on the lawn (10^8^ CFU/plate) of different *Klebsiella spp*. isolates belonging to different clonal lineages and possessing known or unknown capsular types (Supplementary Table 3) were used to estimate host range of the newly isolated bacteriophage. Lysis characteristics were evaluated after 18 h incubation at 37 °C. Genetic determinant of the K24 capsular type was revealed with the following primer pair: K24 Fw: 5′ AGATAATAGGCAACAGCGTTCT 3′ and K24 Rev: 5′ GATACGTTAAACGCCTCAAGTA 3′. Primers were self-designed based on the available sequence data of the *wzi* gene of the *K. pneumoniae* strain 533. These primers were specific to the gene of K24 capsular type and targeted a tyrosine-protein kinase. Validation for positivity was performed on the K24 reference strain CIP 52.229 (Institut Pasteur), and on the strain 533, the sequence of which was confirmed to possess the genetic determinant for the K24 capsule (http://kaptive.holtlab.net/). On the other hand 18 non-K24 capsular type possessing *K. pneumoniae* strains were used as negative controls (Table 1). Conditions of the PCR were as follows: initial denaturation, 95 °C for 2 min; denaturation: 95 °C for 30 s; annealing at 62 °C for 40 s; elongation 72 °C for 1 min; 34 cycles. At the end a one-step termination was applied at 72 °C for 10 min. Primers were evaluated on *K. pneumoniae* 533 and another three − K24 possessing − *K. pneumoniae* strains.

### Efficiency of plating (EOP)

All *K. pneumoniae* isolates sensitive to phage vB_KpnS_Kp13 in the spot test (n = 40) were selected for the determination of EOP as described previously^41^, with some modifications. All bacterial isolates (n = 40) to be tested were grown overnight (18 h) at 37 °C and 100 µl of each culture was used in DLA assay^39^ with 100 µl of diluted phage vB_KpnS_Kp13 lysate. The phage lysate was 10^6^ times diluted from the phage stock. The plates were incubated overnight (18 h) at 37 °C and the number of plaque forming units (PFU) was enumerated. Finally, the EOP was calculated (average PFU on target bacteria / average PFU on host bacteria) along with the standard deviation (± SD) for the three measurements.

The average EOP value for a phage-bacterium ratio was classified according to Mirzaei and Nilsson^41^: highly productive (EOP ≥ 0.5), medium productive (0.1 ≤ EOP < 0.5), low productive (0.001 <EOP < 0.1) or inefficient (EOP ≤ 0.001). The assay was performed in triplicate and results are reported as the mean of three observations (Supplementary Table 1).

### One-step phage growth curve

One-step growth experiments for determination of latent period, log or rise period, plateau phase and burst size was carried out according to a previously published protocol^42^, with some modifications. Five ml of exponential-growth-phase culture of *K. pneumoniae* 533 (10^8^ CFU/ml) and 50 µl of phage suspension (10^7^ PFU/ml) were mixed (MOI 0.1). Phages were allowed to adsorb for 10 min at 37 °C in a shaking incubator (120 RPM), and then the mixture was centrifuged twice at 12,000 RPM for 2 min in order to eliminate the non-adsorbed phages. The pellet was then suspended in 5 ml of fresh LB medium and incubated at 37 °C in a shaking incubator (120 RPM). 200 µl samples were taken at 3 min intervals between 0- and 36-min. Phage titres were estimated by spotting their dilutions on top agar (0.4%) using the DLA method^39^ and incubated overnight (18 h) at 37 °C. The latency period was defined as the time between infection and the shortest incubation time allowing the production of phages^43^. The burst size was calculated as the ratio between the number of phage particles released at the plateau level and the initial number of infected bacterial cells. Experiments were performed three times.

### Killing efficacy of vB_KpnS_Kp13 against *K. pneumoniae* 533 strain and phage-resistant clone screening

Lytic ability of vB_KpnS_Kp13 in different MOIs was demonstrated on exponential-growth-phase cultures of *K. pneumoniae* 533 (10^8^ CFU/ml). A 96-well tissue culture plate (Sarstedt, Germany) was partitioned into 4 parallel sections, each containing 3 columns. Sections 1 and 2 contained 10^5^ CFU/ml (180 μl) of *K. pneumoniae* 533. LB broth without bacteria was used as negative control. Inoculated bacteria without phages served as positive control. All other rows contained 20 μl of bacteriophage suspensions from the phage dilutions of 10^7^ (MOI 100), 10^6^ (MOI 10), 10^5^ (MOI 1), 10^4^ (MOI 0.1), 10^3^ (MOI 0.01), 10^2^ (MOI 0.001) and 10 (MOI 0.0001). Cultures were incubated at 37 °C for 24 h shaking at 180 RPM. Bacterial growth was monitored in a Synergy HT multimode reader (BioTek, Winooski, VT). OD_600_ was measured every 5 min^44^. Experiments were performed in three replicates and reproduced in three independent trials.

The ratio of phage-resistant mutant colonies was assessed by spot testing and liquid culture screening. Phage-resistant clones was assessed on 39 clinical *K. pneumoniae* isolates from the Microbiology Laboratories of the University Hospital in Chester (Supplementary Table 3), through standard spot test^41^. Briefly, 10 µl of the phage dilutions (10^2^, 10^4^, 10^6^) were added to the lawn of *K. pneumoniae* isolates to estimate the phage resistant clones. Following overnight incubation (18 h) at 37 °C, plates were observed for a clear spot in the bacterial lawn. In liquid culture, 3 ml of exponential-growth-phase culture of *K. pneumoniae* 533 (10^8^ CFU/ml) and 3 ml of phage suspensions were mixed at different MOIs (MOI 100, MOI 10, MOI 1, MOI 0.1, MOI 0.01, MOI 0.001 and MOI 0.0001). Cultures were incubated at 37 °C for 7 days, shaking at 120 RPM. 200 µl samples were taken at 24 h intervals. Phage titres and phage resistant isolates were monitored by spot testing.

### Biofilm degradation assay and visualization with CLSM

Biofilm degradation activity of vB_KpnS_Kp13 on the *K. pneumoniae* 533 biofilm was determined using the traditional biofilm assay with slight modifications^45^. Aliquots (200 µl) of *K. pneumoniae* 533 (2 × 10^6^ CFU/ml) were incubated (24 h, 37 °C) in 96-well polystyrene microtitre plates (Sarstedt, Germany). Unattached planktonic cells were carefully removed. Using different MOIs (0.1, 1 and 10) 1 day after biofilm establishment, 200 μl aliquots of vB_KpnS_Kp13 diluted in LB broth were added to each well for 2, 12, 24 and 48 h. LB broth without bacteria was used as a negative control, while inoculated bacteria without phage served as a positive control. The biomass of preformed biofilm was quantified with 1% w/v crystal violet (Sigma, USA) staining for 20 min at RT^46^. After removing the excess dye with PBS, the crystal violet was solubilized in 1% w/v SDS. The absorbance was measured using an ELISA reader (BioRad, USA) at OD_595_. Experiments were performed in triplicate and reproduced in three independent trials.

Structure of *K. pneumoniae* 533 biofilms with or without vB_KpnS_Kp13 biofilm matrix was observed by Confocal Laser Scanning Microscope (CLSM) FV1000 (Olympus Corporation, Japan). The protocol was adapted from a previous publication^47^. Biofilm grew on glass slide pieces (1 × 1 cm) placed in 24-well polystyrene plates (Sarstedt, Germany) at 37 °C for 48 h. The glass pieces were washed with PBS, stained with 0.1% acridine orange (Sigma-Aldrich, Germany) and inspected under CLSM. The biofilm was then infected with vB_KpnS_Kp13 and further incubated for 24 h. The biofilm stack images of slides with vB_KpnS_Kp13 and control biofilm slides without phage infection were analyzed separately using COMSTAT software (MATLAB 5.1, The MathWorks Inc., Natick, MA). The thickness (µm) of the biofilm and biovolume (µm^3^) of bacteria per µm^2^ were recorded.

### Phage genome sequence determination and analysis

Phage DNA was isolated from phage stocks with a concentration ≥10^9^ PFU/ml. Phage DNA was extracted and purified from phage lysate using a QIAGEN Lambda Midi Kit (QIAGEN Inc., CA, USA) and following the manufacturer’s protocol. At the end of the extraction process, DNA samples were dissolved in 100 μl of sterile nuclease free H_2_O. Genomic DNA sequencing libraries were prepared using the Nextera XT Library Preparation kit (Illumina, California, USA). Sequencing was performed using MiSeq Reagent Kit v2 (2 × 150 bp) on an Illumina MiSeq (Illumina, California, USA). Assembly of the pure sequence was performed with the MyPro pipeline^48^. The genome was annotated using the RAST server^49^. After that, the qualified sequence reads were represented using CLC Sequence Viewer v.8 (CLC Bio). Homology searches were conducted with the BLAST tools available at NCBI website (https://www.ncbi.nlm.nih.gov/blast). Identification of open reading frames (ORFs) and gene predictions were used the GeneMark program^50^.

Genome map was illustrated with PHASTER (PHAge Search Tool Enhanced Release, www.phaster.ca).

Function of protein coding sequences (CDSs) in whole phage genome, was predicted by using the UniProtKB database (https://genome.ucsc.edu).

The nucleotide sequence of vB_KpnS_Kp13 was deposited in the GenBank database under the accession number MK170446.

### Phylogenetic analysis

Whole genome based phylogenetic analysis was conducted with VICTOR^51^. All pairwise comparisons of the nucleotide sequence were conducted using the Genome-BLAST Distance Phylogeny (GBDP) method, under settings recommended for prokaryotic viruses. Branch support was inferred from 100 pseudo-bootstrap replicates each. Tree was rooted at the midpoint and visualized with FigTree^52^. Taxon boundaries at the species, genus and family level were estimated with the OPTSIL program with the recommended clustering thresholds and F value (fraction of links required for cluster fusion) of 0.5^51^.

### Rescue experiments in an intraperitoneal (IP) mouse model of infection

For *in vivo* rescue tests, 6–7-week-old (18–21 g) female BALB/c mice (Charles and Rivers, Hungary) were used.

After phage propagation bacterial cells were pelleted with centrifugation. Supernatants were sterile filtered. Phage suspensions were centrifuged (15,000 g, 30 min) and washed 2-times in PBS. As a result, phages became 30x concentrated. PFUs were determined and diluted in order to reach the required 7 × 10^8^ PFU/ml. *K. pneumoniae* suspensions were established from logarithmic cultures. After centrifugation, cells were washed 3-times in PBS and the optical densities were set to OD_600_ = 10 (1 × 10^9^ CFU/ml).

*In vivo* experiments were performed as previously described^53^ with some modifications. Animals were cared for in accordance with the guidelines of the European Federation for Laboratory Animal Science Associations (FELASA) and all procedures, care and handling of the animals were approved by the Animal Welfare Committee of University of Pécs (Permit Number: BA02/2000-37/2015). Five groups (4 mice per group) were used. Four groups were injected intraperitoneally (IP) with 200 µl of *K. pneumoniae* 533 at OD_600_ = 10 (2 × 10^8^ CFU/mice). One group served as a negative or bacteriophage control, receiving 250 µl phage suspensions (1.75 × 10^8^ PFU/mice). The positive control group (only bacterium, no phage) received only 200 µl OD_600_ = 10 bacteria (2 × 10^8^ CFU/mice), while 250 µl bacteriophage suspensions (1.75 × 10^8^ PFU/ mice) were administered to all other groups 10 min, 1 h and 3 h post-infection. General conditions and survival rates of the mice were monitored for 10 days. After 10 days the mice were euthanised by cervical dislocation, lungs and spleen were aseptically removed, and tissue homogenates were subjected to phage count using DLA technique. *K. pneumoniae* 533 was also measured from blood by spot testing. Experiments were performed twice.

### Accession number

The nucleotide sequence of vB_KpnS_Kp13 was deposited in GenBank database under accession number MK170446.

## Supplementary information

Supplementary information
