# Supplementary material for: Identification of a newly isolated lytic bacteriophage against K24 capsular type, carbapenem resistant Klebsiella pneumoniae isolates
Source: Sci Rep. 2020 Apr 3;10:5891. doi: 10.1038/s41598-020-62691-8 (PMC7125228; doi:10.1038/s41598-020-62691-8)
Supplement: Supplementary file 1 — Supplementary information [file 41598_2020_62691_MOESM1_ESM.pdf]

Identification of a newly isolated lytic bacteriophage against K24 capsular type, carbapenem resistant *Klebsiella pneumoniae* isolates

Marianna Horváth[1,5], Tamás Kovács[2], Sarshad Koderivalappil[3], Hajnalka Ábrahám[1], Gábor Rákhely[3,4], György Schneider[5 *]

[1] Department of Medical Biology and Central Electron Microscope Laboratory, University of Pécs, Medical School, Pécs, Hungary.

[2] Department of Biotechnology, Nanophagetherapy Center, Enviroinvest Corporation, Pécs, Hungary.

[3] Department of Biotechnology, University of Szeged, Szeged, Hungary.

[4] Institute of Biophysics, Biological Research Center, Szeged, Hungary.

[5] Department of Medical Microbiology and Immunology, University of Pécs, Medical School, Pécs, Hungary. * Correspondence to schneider.gyorgy@pte.hu.

Supplementary Figure 1.

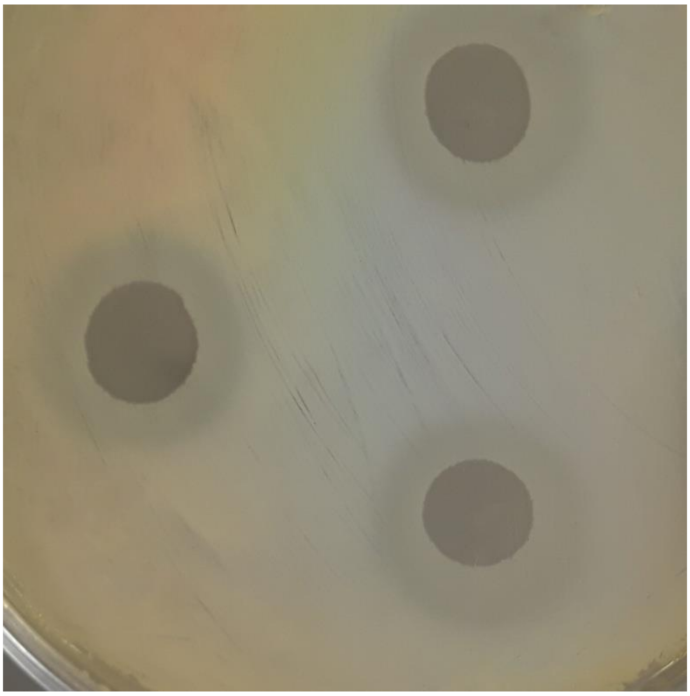

Supplementary Figure 1. Plaque morphology of phage vB_KpnS_Kp13. Clear plaques (~10 mm) surrounded by a wide halo zone (~4 mm) formed by vB_KpnS_Kp13 with *K. pneumoniae* 533 lawn on double layer agar plate.

Supplementary Table 1. Efficiency of plating (EOP) for phage vB_KpnS_Kp13 on *K. pneumoniae* isolates to the phage infection. For each isolate, the strain name, the sequence type, EOP value and EOP classification are reported. The type of phage production is ranked according to Mirzaei and Nilsson (Mirzaei & Nilsson, 2015). Each EOP value is the mean of three observations ±SD.

Strain	Sequence type	EOP value	Production
**101**	ST15	0.5±0.2	High
**104**	ST15	0.5±0.3	High
**106**	ST15	0.6±0.2	High
**111**	ST15	0.8±0.2	High
**113**	ST15	0.5±0.3	High
**531**	ST15	0.5±0.3	High
**532**	ST15	0.5±0.3	High
**533**	ST15	1±0.2	High
**534**	ST15	0.8±0.7	High
**535**	ST15	0.7±0.4	High
**536**	ST15	0.6±0.2	High
**538**	ST15	0.5±0.3	High
**539**	ST15	0.5±0.2	High
**5310**	ST15	0.4±0.2	Medium
**5311**	ST15	0.6±0.2	High
**5313**	ST15	0.5±0.3	High
**501**	ST15	0.5±0.2	High
**502**	ST15	0.7±0.2	High
**503**	ST15	0.6±0.3	High
**I1**	ST15	0.5±0.2	High
**491**	ST15	0.6±0.3	High
**492**	ST15	0.6±0.2	High
**493**	ST15	0.7±0.4	High
**C614**	-	0.6±0.3	High
**C715**	ST15	0.5±0.2	High
**C815**	ST15	0.5±0.2	High
**C1015**	ST15	0.4±0.3	Medium
**C1115**	ST15	0.6±0.2	High
**C1215**	ST15	0.7±0.4	High

Strain	Sequence type	EOP value	Production
**C1315**	ST15	0.6±0.3	High
**C1415**	ST15	0.5±0.2	High
**C1515**	ST15	0.6±0.2	High
**C1615**	ST15	0.6±0.4	High
**C1715**	ST15	0.5±0.3	High
**C1815**	-	0.5±0.2	Medium
**C116**	ST15	0.7±0.2	High
**C217**	-	0.6±0.4	High
**C317**	-	0.5±0.3	High
**C517**	-	0.5±0.2	High
**CIP 52.229**	ST59	0.6±0.2	High

Supplementary Table 2. The COMSTAT analysis of the surface plots. The thickness (µm) of the biofilms and the biovolume (µm^3) of bacteria per µm^2 of the surface were recorded.

**Parameters**	**Control *K. pneumoniae* 533 (µm)**			**Average (µm)**	**vB_KpnS_Kp13 treated (µm)**			**Average (µm)**
**Biomass (µm^3/µm^2)**	13.02	12.96	13.39	**13.12**	5.02	4.56	4.70	**4.76**
**Average thickness (µm)**	18.13	19.20	18.41	**18.58**	9.03	9.90	8.80	**9.24**
**Maximum thickness (µm)**	23.01	22.60	23.66	**23.09**	16.06	15.09	16.03	**15.73**

Supplementary Table 3. Characteristics of the bacterial strains used for determination of phage vB_KpnS_Kp13 bacterial host range.

Strain	Bacteria	Isolated from	Sequence type	K-serotype	Lysis	Reference/Source
**NTUH-K2044**	*K. pneumoniae*	blood	ST23	K1	−	NTUH-K2044
**CIP 52.145**	*K. pneumoniae*	-	ST66	K2	−	Institut Pasteur*
**CIP 80.51**	*K. pneumoniae subsp. rhinoscleromatis*	-	-	K3	−	Institut Pasteur*
**ATCC 700603**	*K. quasipneumoniae*	urine	ST489	K6	−	ATCC 700603
**CIP 52.207**	*K. pneumoniae*	-	-	K9	−	Institut Pasteur*
**CIP 52.215**	*K. pneumoniae*	-	-	K11	−	Institut Pasteur*
**CIP 52.216**	*K. pneumoniae*	-	-	K12	−	Institut Pasteur*
**CIP 52.217**	*K. pneumoniae*	-	-	K13	−	Institut Pasteur*
**CIP 52.221**	*K. pneumoniae*	-	-	K17	−	Institut Pasteur*
**CIP 52.223**	*K. pneumoniae*	-	-	K19	−	Institut Pasteur*
**CIP 52.224**	*K. pneumoniae*	-	-	K20	−	Institut Pasteur*
**CIP 52.225**	*K. pneumoniae*	-	-	K21	−	Institut Pasteur*
**CIP 52.229**	*K. pneumoniae*	-	ST59	K24	+	Institut Pasteur*
**101**	*K. pneumoniae*	urine	ST15	K24	+	Clinical isolate, CCH**
**104**	*K. pneumoniae*	urine	ST15	K24	+	Clinical isolate, CCH**
**106**	*K. pneumoniae*	urine	ST15	K24	+	Clinical isolate, CCH**
**111**	*K. pneumoniae*	urine	ST15	K24	+	Clinical isolate, CCH**
**113**	*K. pneumoniae*	urine	ST15	K24	+	Clinical isolate, CCH**
**531**	*K. pneumoniae*	faeces, urine, sputum	ST15	K24	+	Clinical isolate, CCH**
**532**	*K. pneumoniae*	faeces, urine	ST15	K24	+	Clinical isolate, CCH**
**533**	*K. pneumoniae*	urine	ST15	K24	+	Clinical isolate, CCH**
**534**	*K. pneumoniae*	faeces, sputum	ST15	K24	+	Clinical isolate, CCH**
**535**	*K. pneumoniae*	faeces, sputum, blood	ST15	K24	+	Clinical isolate, CCH*
**536**	*K. pneumoniae*	faeces	ST15	K24	+	Clinical isolate, CCH**
**538**	*K. pneumoniae*	urine	ST15	K24	+	Clinical isolate, CCH**
**539**	*K. pneumoniae*	faeces	ST15	K24	+	Clinical isolate, CCH**
**5310**	*K. pneumoniae*	faeces	ST15	K24	+	Clinical isolate, CCH**

Strain	Bacteria	Isolated from	Sequence type	K-serotype	Lysis	Reference/Source
**5311**	*K. pneumoniae*	faeces, urine	ST15	K24	+	Clinical isolate, CCH**
**5313**	*K. pneumoniae*	faeces	ST15	K24	+	Clinical isolate, CCH**
**501**	*K. pneumoniae*	feces, blood	ST15	K24	+	Clinical isolate, CCH**
**502**	*K. pneumoniae*	sputum	ST15	K24	+	Clinical isolate, CCH**
**503**	*K. pneumoniae*	faeces	ST15	K24	+	Clinical isolate, CCH**
**I1**	*K. pneumoniae*	faeces	ST15	K24	+	Clinical isolate, CCH**
**491**	*K. pneumoniae*	faeces	ST15	K24	+	Clinical isolate, CCH**
**492**	*K. pneumoniae*	faeces	ST15	K24	+	Clinical isolate, CCH**
**493**	*K. pneumoniae*	faeces	ST15	K24	+	Clinical isolate, CCH**
**C614**	*K. pneumoniae*	faeces	-	K24	+	Clinical isolate, CCH**
**C715**	*K. pneumoniae*	faeces	ST15	K24	+	Clinical isolate, CCH**
**C815**	*K. pneumoniae*	faeces, urine	ST15	K24	+	Clinical isolate, CCH**
**C1015**	*K. pneumoniae*	faeces	ST15	K24	+	Clinical isolate, CCH**
**C1115**	*K. pneumoniae*	faeces	ST15	K24	+	Clinical isolate, CCH**
**C1215**	*K. pneumoniae*	urine	ST15	K24	+	Clinical isolate, CCH**
**C1315**	*K. pneumoniae*	faeces	ST15	K24	+	Clinical isolate, CCH**
**C1415**	*K. pneumoniae*	faeces, urine	ST15	K24	+	Clinical isolate, CCH**
**C1515**	*K. pneumoniae*	urine	ST15	K24	+	Clinical isolate, CCH**
**C1615**	*K. pneumoniae*	faeces	ST15	K24	+	Clinical isolate, CCH**
**C1715**	*K. pneumoniae*	faeces	ST15	K24	+	Clinical isolate, CCH**
**C1815**	*K. pneumoniae*	urine	-	K24	+	Clinical isolate, CCH**
**C116**	*K. pneumoniae*	faeces, urine	ST15	K24	+	Clinical isolate, CCH**
**C217**	*K. pneumoniae*	faeces, urine	-	K24	+	Clinical isolate, CCH**
**C317**	*K. pneumoniae*	faeces	-	K24	+	Clinical isolate, CCH**
**C517**	*K. pneumoniae*	faeces, blood	-	K24	+	Clinical isolate, CCH**
**NPHMOS_9**	*K. pneumoniae*	faeces	-	not K24	−	Clinical isolate, own collection
**NPHMOS_25**	*K. pneumoniae*	faeces	-	not K24	−	Clinical isolate, own collection
**NPHMOS_44**	*K. pneumoniae*	faeces	-	not K24	−	Clinical isolate, own collection
**NPHMOS_69**	*K. pneumoniae*	faeces	-	not K24	−	Clinical isolate, own c.

Strain	Bacteria	Isolated from	Sequence type	K-serotype	Lysis	Reference/Source
**NPHMOS_88**	*K. pneumoniae*	faeces	-	not K24	–	Clinical isolate, own collection
**Sew_4**	*K. pneumoniae*	sewage	-	not K24	–	Sewage isolate, own coll
**Sew_19**	*K. pneumoniae*	sewage	-	not K24	–	Sewage isolate, own collection (Pellérd)
**Sew_33**	*K. pneumoniae*	sewage	-	not K24	–	Sewage isolate, own collection (Pellérd)
**Sew_55**	*K. pneumoniae*	sewage	-	not K24	–	Sewage isolate, own collection (Pellérd)
**Sew_72**	*K. pneumoniae*	sewage	-	not K24	–	Sewage isolate, own collection (Pellérd)
**KP2201**	*K. pneumoniae*	faeces	-	not K 24	–	Clinical isolate, own collection
**KP2244**	*K. pneumoniae*	faeces	-	not K24	–	Clinical isolate, own collection
**KP2254**	*K. pneumoniae*	faeces	-	not K24	–	Clinical isolate, own collection
**KP2266**	*K. pneumoniae*	faeces	-	not K24	–	Clinical isolate, own collection
**KP2294**	*K. pneumoniae*	faeces	-	not K24	–	Clinical isolate, own collection
**HC11**	*K. pneumoniae*	blood	-	not K24	–	Clinical isolate, own collection
**HC372**	*K. pneumoniae*	blood	-	not K24	–	Clinical isolate, own collection
**HC494**	*K. pneumoniae*	blood	-	not K24	–	Clinical isolates, own collection
**HC899**	*K. pneumoniae*	blood	-	not K24	–	Clinical isolate, own collection
**HC998**	*K. pneumoniae*	blood	-	not K24	–	Clinical isolate, own collection
**U43**	*K. pneumoniae*	urine	-	not K24	–	Clinical isolate, own
**U210**	*K. pneumoniae*	urine	-	not K24	–	Clinical isolate, own collection

Strain	Bacteria	Isolated from	Sequence type	K-serotype	Lysis	Reference/Source
**U490**	*K. pneumoniae*	urine	-	not K24	−	Clinical isolate, own collection
**U655**	*K. pneumoniae*	urine	-	not K24	−	Clinical isolate, own collection
**U820**	*K. pneumoniae*	urine	-	not K24	−	Clinical isolate, own collection
**E1191**	*K. pneumoniae*	faeces	-	not K24	−	Clinical isolate, own collection
**E1496**	*K. pneumoniae*	faeces	-	not K24	−	Clinical isolate, own collection
**E1531**	*K. pneumoniae*	faeces	-	not K24	−	Clinical isolate, own collection
**E1540**	*K. pneumoniae*	faeces	-	not K24	−	Clinical isolate, own collection
**E1779**	*K. pneumoniae*	faeces	-	not K24	−	Clinical isolate, own collection
**CIP 52.232**	*K. pneumoniae*	-	-	K27	−	Institut Pasteur*
**CIP 52.235**	*K. pneumoniae*	-	-	K30	−	Institut Pasteur*
**CIP 53.8**	*K. pneumoniae*	-	-	K33	−	Institut Pasteur*
**CIP 53.23**	*K. pneumoniae*	-	-	K47	−	Institut Pasteur*
**MGH 78578**	*K. pneumoniae*	sputum	ST38	K52	−	MGH 78578
**CIP 80.47**	*K. pneumoniae*	-	-	K64	−	Institut Pasteur*

* Institut Pasteur, 25-28 rue du Dr. Roux, Paris, France.

**CCH: Countess of Chester Hospital, Department of Microbiology, Chester, Cheshire, UK.
